# Developing a Multi-Layer Deep Learning Based Predictive Model to Identify DNA N4-Methylcytosine Modifications

**DOI:** 10.3389/fbioe.2020.00274

**Published:** 2020-04-21

**Authors:** Rao Zeng, Minghong Liao

**Affiliations:** Department of Software Engineering, School of Informatics, Xiamen University, Xiamen, China

**Keywords:** DNA N4-methylcytosine, deep learning, site prediction, webserver, feature representation

## Abstract

DNA N4-methylcytosine modification (4mC) plays an essential role in a variety of biological processes. Therefore, accurate identification the 4mC distribution in genome-scale is important for systematically understanding its biological functions. In this study, we present Deep4mcPred, a multi-layer deep learning based predictive model to identify DNA N4-methylcytosine modifications. In this predictor, we for the first time integrate residual network and recurrent neural network to build a multi-layer deep learning predictive system. As compared to existing predictors using traditional machine learning, our proposed method has two advantages. First, our deep learning framework does not need to specify the features when training the predictive model. It can automatically learn the high-level features and capture the characteristic specificity of 4mC sites, benefiting to distinguish true 4mC sites from non-4mC sites. On the other hand, our deep learning method outperforms the traditional machine learning predictors in performance by benchmarking comparison, demonstrating that the proposed Deep4mcPred is more effective in the DNA 4mC site prediction. Moreover, via experimental comparison, we found that attention mechanism introduced into the deep learning framework is useful to capture the critical features. Additionally, we develop a webserver implementing the proposed method for the academic use of research community, which is now available at http://server.malab.cn/Deep4mcPred.

## Introduction

Epigenetics refers to the heritable phenotype changes in the function of genes that do not involve alterations in DNA sequence. DNA methylation refers to the binding of a methyl group on the nucleotide of DNA (Liu et al., 2019a) under the action of DNA methyltransferases (Dnmt). As one of the earliest discovered and most in-depth epigenetic regulation mechanisms, it is associated with normal development and plays an essential role in key biological processes including regulating gene expression, regulating mammalian growth and development, mediating X chromosome inactivation, and participating in gene imprinting (Jin et al., 2011). It can be divided into three categories according to the position of methylation modification: N6-methyladenine (6mA), 5-Methylcytosine (5mC) and N4-methylcytosine (4mC) (Chen et al., 2017; Wei et al., 2019a). The most prevalent methylation modification in eukaryotes is 5mC (Luo et al., 2015; Xiao et al., 2018) which consists of methylation at the fifth position of the cytosine pyrimidine ring and has focused on epigenetic markers in mammals and plants (Liu et al., 2019a), while 6mA (methylations on the sixth position of the adenine purine ring) (Liu et al., 2019a) is the most predominant DNA modification in prokaryote and has been found to be related to the regulation of restriction-modification (R-M) system, DNA mismatch repair, gene expression, and other aspects (Luo et al., 2015; Xiao et al., 2018). With the development of high-throughput techniques, the 4mC (methylations on the fourth position of the cytosine pyrimidine ring) was discovered in bacteria, and found to play an important role in protecting genome from invasion in restriction-modification (R-M) system. Developing methods to explore more biological functions of 4mC is of significance.

Single-molecule real time sequencing (SMRT) technology has been proposed to detect the 4mC and 6mA sites from the whole genome (Flusberg et al., 2010). However, using SMRT techniques to analyze the genome is costly inefficient. Therefore, Yu et al. (2015) proposed 4mC-Tet-assisted bisulfite-sequencing (4mC-TABseq) as a new generation of sequencing technology (Illumina sequencing systems) to identify the genome-wide locations of 4mC for bacterial species more rapidly and cost efficiently. Although the prediction of 4mC sites by this sequencing technique has been improved to some extent, recent studies focus more on the recognition of 4mC sites using machine learning, which is capable of predicting 4mC sites based on genome sequences, without any prior experimental knowledge. There are currently four methods available in literature to identify 4mC sites, including iDNA4mC (Chen et al., 2017), 4mCPred (Su et al., 2018), 4mcPred-SVM (Wei et al., 2018a), and 4mcPred-IFL (Wei et al., 2019a). iDNA4mC, as the first machine learning predictor, encodes sequences by nucleotide chemical properties and nucleotide frequency to features and trains support vector machine (SVM) models for prediction (Liang et al., 2018). Although this method has the ability to distinguish between 4mC and non-4mC sites, the prediction accuracy is relatively low overall. Afterwards, He et al. proposed 4mCPred, an SVM-based predictive model trained with position-specific trinucleotide propensity (PSTNP) and electron-ion interaction potential features. More recently, 4mcPred-SVM and 4mcPred-IFL, proposed by Wei et al., further improve the predictive performance on the same golden benchmark datasets. The former employs a two-step feature optimization strategy to improve the feature representation ability, while the latter uses an iterative feature representation algorithm to learn critical information from several sequential feature models. Even though the above methods have improved the performance for identifying 4mC sites, too few data sets have been adopted to fully reflect the whole genome and to build robust models. Consequently, it is eager and indispensable to develop a robust and strong model to more accurately identify 4mC sites.

In recent years, deep learning is not only developed as a new research direction in machine learning, but also has made a lot of achievements in data mining (Lan et al., 2018), speech recognition (Amodei et al., 2014), machine translation (Sutskever et al., 2014), natural language processing (Collobert and Weston, 2008; Young et al., 2018), and other related fields (Hong et al., 2019; Li and Liu, 2019; Liu et al., 2019b; Yang et al., 2019; Zeng et al., 2019a,b). In the field of computational biology, deep learning has been widely applied, especially in solving the problems of genome sequence-based by convolutional neural networks (CNN) (Nie et al., 2018; Peng et al., 2018; Lv et al., 2019a; Wang et al., 2019; Zhang et al., 2019a; Zou et al., 2019). In this paper, we proposed Deep4mcPred, a multi-layer deep learning based predictive model to identify DNA N4-methylcytosine modifications. In this predictor, we for the first time integrate residual network (He et al., 2016) and recurrent neural network, together with attention mechanism, to build a multi-layer deep learning predictive system. We evaluated and compared our predictor with existing predictors. The comparative results demonstrate that our proposed model can more accurately identify 4mC sites than the state-of-the-art predictors. In addition, the proposed method is implemented by the simple and easy-to-use webserver which is freely available on http://server.malab.cn/Deep4mcPred.

## Methods and Materials

### Dataset Collection

Previous study has demonstrated that a stringent dataset is essential for building a robust predictive model (Zeng et al., 2016, 2017a; Liu et al., 2017; Wei et al., 2017a, 2018b,c; Jin et al., 2019; Liu, 2019; Su et al., 2019). In existing studies, there is one golden benchmark dataset proposed by Chen et al. for performance evaluation and comparison. However, the size of the dataset is too small to train a deep learning model. Accordingly, we constructed a larger dataset in this study. We strictly followed the data processing procedure as introduced in Chen's study. By doing so, we can guarantee our dataset the most representative.

#### Positive Samples Collection

Specifically, there are three main steps for collecting the positive samples. Firstly, we collected all 41bp long sequences centered with true 4mC sites from the MethSMRT database (Ye et al., 2016). Next, we removed the sequences with Modification QV (modQV) score not <30 as it is the default threshold for invoking the modification location according to the Methylome Analysis Technical Note. Next, we used CD-HIT software (with the threshold of 80%) (Fu et al., 2012) to reduce the identity of the positives, avoiding the potential of performance biased-estimation. Ultimately, following the procedure, we collected the positive samples from three species: *Arabidopsis thaliana* (*A. thaliana*), *Caenorhabditis elegans* (*C. elegans*), and *Drosophila melanogaster* (*D. melanogaster*). The details of the positive samples in the three species are presented in Table 1. Note that we randomly picked 20,000 positive samples for model training.

**Table 1:** Summary of benchmark datasets in three species.

**Species**	**Positives**	**Negatives**	**Total**
*A. thaliana*	20,000	20,000	40,000
*C. elegans*	20,000	20,000	40,000
*D. melanogaster*	20,000	20,000	40,000

#### Negative Samples Collection

The negative samples were also cytosine-centered sequences with a length of 41bp but are not recognized by the SMRT sequencing technology. In this case, the number of negative samples per species are much larger than the corresponding positive samples. To avoid the data imbalance problem, we randomly selected the same number of negative samples with that of the positive samples in corresponding species for model training.

### The Framework of the Proposed Deep Learning Method

**Figure 1:**
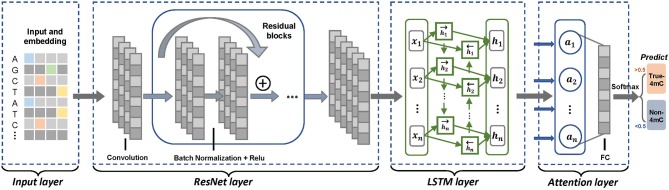
The illustration of the deep neural network. It is a four-layer prediction system. Firstly, given sequences are fed to the input layer for feature representation using one-hot encoding, thus generating feature matrixes. Next, we feed the matrixes to the ResNet layer for extracting global features. After that, in LSTM layer, we use bidirectional LSTM model to gather feature information from two directions. In Attention layer can learn more relevant feature information. Ultimately, the features are connected with full connected layer and Softmax can make predictions. If the prediction score is higher than 0.5, the predicted sequences are 4mc sequences; otherwise, they are not.

Figure 1 illustrates the overall predictive framework of the proposed multi-layer deep learning network. For given DNA sequences, neural network is composed of four layers: the input layer, the ResNet layer, the LSTM layer and the attention layer, as seen in Figure 1. The first layer is the input layer. The sequences of the dataset are encoded by one-hot method and the obtained features are fed into the subsequent ResNet layer. Through this residual network model, deeper networks can be built than plain CNN models for extracting effective global features. The output feature vectors are utilized as inputs of the LSTM layer. In the LSTM layer, the bidirectional LSTM model is utilized to gather feature information from two directions which has been proven to be more effective than the unidirectional LSTM model. In the last attention layer, the attention mechanism is introduced to integrate the output of the LSTM layer for more relevant feature information. Finally, a fully-connected neural network (FC) is attached after the attention model and the softmax activation function is performed to make predictions.

### Sequence Representation Using One-Hot Encoding

Genomic sequences are consisting of four nucleotides: “A” (adenine), “G” (guanine), “C” (cytosine), and “T” (thymine). Undetermined bases are annotated as “N.” The nucleotides are represented using one-hot encoding over four bits. For example, “A” is represented as the binary vector (1,0,0,0); “G” is encoded as (0,1,0,0); “C” is encoded as (0,0,1,0); “T” is encoded as (0,0,0,1); and “N” is (0,0,0,0).

### Deep Learning Model Architecture

We developed a novel prediction method, namely Deep4mcPred, that integrates Long Short Term Memory (LSTM) recurrent neural network and the attention mechanism into the Residual Networks (ResNet). The overall architecture of our proposed model is shown in Figure 1.

#### Residual Networks (ResNet)

Studies have showed that the overall performance of the network is greatly affected by the number of network layers when it comes to convolutional neural network (CNN). To be specific, the accuracy of the network increases as the depth increases, but when the depth reaches a certain level, the accuracy begins to drop rapidly. This is called the degradation problem, making it difficult to generate very deep neural networks.

To address this, ResNet introduces a residual learning framework to improve the degradation, which has achieved great success in the areas of image classification and item identification in recent studies. The internal residual blocks of ResNet utilize jump connections, alleviating the problem of gradient disappearance caused by the increase of depth in convolutional neural networks.

For an input x, ResNet learns a specific residual function *F*(*x*) = *H*(*x*) − *x*, whereas *F*(*x*) = *H*(*x*) for plain CNN. Supposing the residual *F*(*x*) = 0, then it occurs identity mapping “shortcut.” The residual block is performed as follows:

$$\begin{array}{l} y=F\left(X,\left\{W_{i}\right\}\right)+x \end{array}$$

where

$$\begin{array}{l} F=W_{2}\sigma \left(x,\;W_{i}\right) \end{array}$$

where the function *F* denotes the learned residual mapping and σ represents relu. *F* and *x* are added element by element under the premise of shortcut connections.

But in fact, the residual *F*(*x*) will not be zero, so the dimensions of *F* and *x* will be different. The output of the ResNet layer can be formulated as follows:

$$\begin{array}{l} y=F\left(X,\left\{W_{i}\right\}\right)+W_{s}x \end{array}$$

where *W*_*s*_ is introduced to perform a linear mapping to match the dimensions. Taking consider of ResNet, it allows the stacked layer to extract more distinct features of the input x, resulting in better performance.

#### Long Short Term Memory (LSTM)

Recurrent Neural Network (RNN) is a powerful neural network for processing sequential data. The parameter learning of the RNN is performed by the back-propagation algorithm over time. When the input sequence is long, a gradient disappearance or gradient explosion problem occurs, which is termed as long-term dependency problem.

LSTM is one type of RNN, which introduces the conception of self-loop to generate a path of continuous gradient flow for a long time and gating mechanism to control the information flow, solving the long-term dependency problem. It was firstly proposed by Hochreiter and Schmidhuber in 1997. From then on, LSTM has achieved considerable success and has been widely used in the fields of handwriting recognition, machine translation, and speech recognition, etc.

The stacked architecture of LSTM is shown in Figure 1. The output from the ResNet layers is fed into the subsequent LSTM layer as the input. Then, the LSTM components are updated by the following formulations:

$$\begin{array}{l} \left(\begin{array}{c} i_{t}\\ f_{t}\\ C_{t}^{\prime } \end{array}\right)=\left(\begin{array}{c} \sigma \\ \sigma \\ tanh \end{array}\right)\left(\left(\begin{array}{c} W_{x}\\ W_{h}\\ W_{c} \end{array}\right)\left[h_{t-1},x_{t},c_{t-1}\right]+\left(\begin{array}{c} b_{i}\\ b_{f}\\ b_{g} \end{array}\right)\right)\\ \begin{array}{l} \;C_{t}=i_{t}C_{\;t}^{\prime }\text{ + }f_{t}C_{t-1}\\ \;o_{t}=\sigma \left(W_{x}h_{t-1}+W_{h}x_{t}+W_{c}C_{t}+b_{o}\right)\\ \;h_{t}=o_{t}\tanh\left(C_{t}\right) \end{array} \end{array}$$

where *i*_*t*_, *f*_*t*_ and *o*_*t*_ represent the input, forget and output gate, respectively; $C_{\;t}^{\prime }$ is an auxiliary value for calculating the cell memory *C*_*t*_; *t* denotes the recurrent time step; *W*_*x*_, *W*_*h*_, *W*_*c*_, and *b* are the corresponding weight values for each equation; and the current output of LSTM cell is *h*_*t*_ at time step *t*.

In consideration of bidirectional LSTM, the final LSTM network is composed of two LSTM networks with opposite directions. Hence, the i-th deoxynucleotide of the DNA sequence can be encoded as below:

$$\begin{array}{l} h_{i}=\left[\vec{h_{i}}⊕\overset{⃖}{h_{i}}\right] \end{array}$$

#### Attention Mechanism

Inspired by human attention, the attention mechanism is an idea for solving problems that focuses on the important factors while ignoring the unimportant. The attention mechanism can quickly filter out high-level information from noises, which has recently demonstrated great success in many relevant classification tasks. To take advantage of this, we applied the attention mechanism after the LSTM layer in the model to obtain the final distinctive feature representation. Let *H* be the output vectors [*h*_1_, *h*_2_, …, *h*_*s*_] generated by LSTM layer, where *s* is the length of the DNA sequence. As shown in Figure 1, the following formulations are performed in the attention layer:

$$\begin{array}{r} M=\tanh\left(H\right)\\ \alpha =softmax\left(W^{T}M\right)\\ r=H\alpha^{T} \end{array}$$

where *W*^*T*^ is a transpose of the trained parameter vector *W*. Then the final representation of the attention layer can be encoded as below:

$$\begin{array}{l} h^{*}=\tanh\left(r\right) \end{array}$$

#### Softmax

The generated vectors h^*^ after the attention module are fed into a softmax layer for classification as input. The softmax score of class *k* will be calculated as follows:

$$\begin{array}{l} \alpha_{k}=\frac{e^{h^{*}}}{\sum_{k=1}^{C}e^{h^{*}}} \end{array}$$

where *C* denotes the total number of categories, and *C* = 2 when dealing with the binary classification tasks.

The softmax function maps and the output of neurons to numbers between (0–1) and normalizes the sum to 1. In other words, the output scores of each category can be converted into a relative probability by softmax. Therefore, the predicted label can be determined by comparing the predicted probability α_*k*_ for each class.

At last, we generated a multi-layered neural network integrating ResNet with a LSTM layer and an attention module, which incorporates the strengths behind ResNet, LSTM, and the attention mechanism. Through applying such a comprehensive network structure, feature extraction and learning are combined in an end-to-end manner, which can significantly improve the prediction performance.

### Performance Indicators

In our experiment, we used the following four indicators to evaluate the predictive performance of our proposed model, including Accuracy (ACC), Sensitivity (SN), Specificity (SP), and Mathew's Correlation Coefficient (MCC). They are the four commonly used indicators for classifier performance evaluation in other Bioinformatics fields (Zhang et al., 2008, 2018a,b,c, 2019b,c,d; Wei et al., 2017b, 2019b; Zeng et al., 2017b, 2019c; Chen et al., 2018; Lu et al., 2018a,b; Fu et al., 2019; Gong et al., 2019; Jin et al., 2019; Liu and Li, 2019; Liu et al., 2019c,d; Manavalan et al., 2019a,b,c,d; Basith et al., 2020). Their calculation formulas are as follows:

$$\begin{array}{l} \{\begin{array}{c} Sn=\frac{TP}{TP+FN}\;0\leq Sn\leq 1\\ Sp=\frac{TN}{TN+FP}\;0\leq Sp\leq 1\\ ACC=\frac{TP+TN}{TP+FP+TN+FN}\;0\leq ACC\leq 1\\ MCC=\frac{TP\times TN-FP\times FN}{\sqrt{\left(TN+FN\right)\times \left(TN+FP\right)\times \left(TP+FN\right)\times \left(TP+FP\right)}}\;-1\leq MCC\leq 1 \end{array} \end{array}$$

where TP (True Positive) represents the number of positive samples correctly predicted; TN (True Negative) represents the number of negative samples correctly predicted; FP (False Positive) represents the number of negative samples incorrectly predicted to be the positives; FN (False Negative) represents the number of positive samples incorrectly predicted to be the negatives.

Moreover, we also used the area under the ROC curve (AUC) is to quantitively measure the predictive performance of the model (Yang et al., 2018; Lv et al., 2019b; Niu et al., 2019). A higher AUC represents a better predictor (Hanley and McNeil, 1982; Liu et al., 2018; Feng et al., 2019; Lai et al., 2019).

## Results and Discussions

### Comparison of the Proposed Method and Existing Predictors

To examine the predictive performance of our deep learning model, we compared several existing predictors with our model, including iDNA4mC (Chen et al., 2017), 4mCPred (Su et al., 2018), 4mcPred-SVM (Wei et al., 2018a), and 4mcPred-IFL (Wei et al., 2019a). It is worth noting that besides our predictor using deep learning, other compared predictors are all traditional machine learning algorithm -SVM and different handcrafted sequential features to train their respective models. For fair comparison, all the predictors are evaluated with 10-fold cross validation on the same dataset used in this study.

Table 2 lists the performances of the proposed method and four existing predictors. We can see that our proposed deep learning method achieves the highest performance in two out of three species (*C. elegans* and *A. thaliana*), with only one exception in *D. melanogaster*, in which our method is slightly worse than existing predictors. Specifically, for *C. elegans*, our predictor achieves 91.5%, 87.2%, 89.3%, and 0.787 in terms of SN, SP, ACC, and MCC, respectively. The overall performances (ACC and MCC) by our predictor are significantly better than the runner-up predictor−4mcPred-IFL (with the ACC of 88.0% and the MCC of 0.761). The more significant improvement is observed in *A. thaliana*, in which our predictor outperforms existing predictors in all metrics, leading by 5.7%, 2.2%, and 0.045 in terms of SN, ACC, and MCC, respectively. In addition, we found that our model remarkably improves the SN in all three species, demonstrating that our deep learning model can more accurately identify true 4mC sites. To better illustrate the difference between various models, we used Delong's test from the R package pROC to compare the ROC curves, confirming that the performance gain from fixed-length to full-length version is statistically significant (*p* = 0.0005). Generally, the comparative results demonstrate that our deep learning model is better than existing predictors using traditional machine learning algorithms in prediction of 4mC sites. More importantly, our deep learning model can automatically learn high-level feature representations to capture the characteristics of 4mC sites, rather than specify sequence-based features before model training as existing predictors did.

**Table 2:** Performance comparison of the proposed Deep4mcPred and existing sequence-based predictors.

**Species**	**Predictors**	**SN (%)**	**SP (%)**	**ACC (%)**	**MCC**
*C. elegans*	iDNA4mc	79.0	77.0	78.0	0.560
	4mcPred	82.5	82.6	82.6	0.652
	4mcPred_SVM	82.4	80.7	81.5	0.631
	4mcPred_IFL	89.0	87.1	88.0	0.761
	**Deep4mcPred**	**91.5**	**87.2**	**89.3**	**0.787**
*D. melanogaster*	iDNA4mc	83.3	79.0	81.2	0.620
	4mcPred	82.4	82.1	82.2	0.646
	4mcPred_SVM	83.8	82.2	83.0	0.661
	4mcPred_IFL	86.5	88.0	87.3	0.745
	**Deep4mcPred**	**87.6**	**86.6**	**87.1**	**0.742**
*A. thaliana*	iDNA4mc	76.6	75.5	76.1	0.520
	4mcPred	75.5	78.0	76.8	0.536
	4mcPred_SVM	77.8	79.6	78.7	0.573
	4mcPred_IFL	80.3	84.0	82.2	0.644
	**Deep4mcPred**	**86.0**	**82.9**	**84.4**	**0.689**

*The performances are evaluated with 10-fold cross validation. Note that the performances of the other methods are cited from existing studies, since the source codes of existing methods are not available. The value in bold indicates the optimal value of the indicator*.

### Performance Impact by Integrating Attention Mechanism

In this section, we evaluated whether or not the attention mechanism can improve the performance of 4mC site prediction. Subsequently, we compared the models taking into account attention mechanism and the model not taking into account attention mechanism for prediction. Both models were trained and evaluated with 10-fold cross validation on the dataset used in this study.

**Figure 2:**
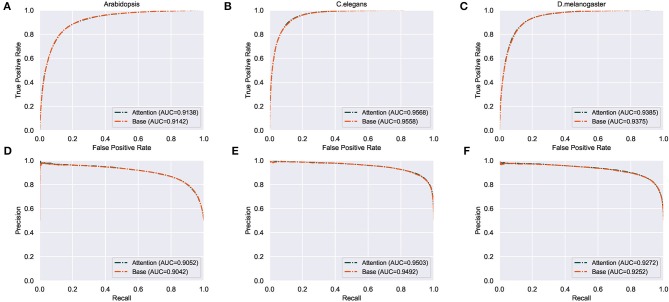
Performance of the model using the attention mechanism and the model not using the attention mechanism. **(A–C)** represent the ROC curves of the two models in the three species. **(D–F)** represent the PR curves of the two models in the three species, respectively.

Results in Table 3 show that training with the attention mechanism, the model achieves 89.3% in ACC and 0.787 in MCC for *C. elegans* dataset, achieves 87.1% in ACC and 0.742 in MCC for the *D. melanogaster* dataset, achieves 84.4% in ACC and 0.689 in MCC for the *A. thaliana* dataset, respectively. These results demonstrate that using the attention mechanism we can achieve good performances for 4mC sites prediction for different species. The comparison between the models using and not using the attention mechanism is shown in Figure 2. We can observe that the model using attention mechanism performs better than the model not using the attention mechanism in ROC and PR curves. The details of the performances for both models are listed in Table 3. Results show that using the attention mechanism, the deep learning model can achieve the average improvement of 0.1% roughly in three species as compared to the model not using the attention mechanism. This demonstrates that the attention mechanism indeed helps to capture discriminative feature representations.

**Table 3:** Performance comparison of the model using the attention mechanism and the model not using the attention mechanism.

**Species**	**Models**	**SN (%)**	**SP (%)**	**ACC (%)**	**MCC**
*C. elegans*	ResNet_LSTM_Attention	91.5	87.2	89.3	0.787
	ResNet_LSTM	90.9	87.2	89.0	0.781
*D. melanogaster*	ResNet_LSTM_Attention	87.6	86.6	87.1	0.742
	ResNet_LSTM	87.7	86.3	87.0	0.740
*A. thaliana*	ResNet_LSTM_Attention	86.0	82.9	84.4	0.689
	ResNet_LSTM	84.9	83.9	84.4	0.688

## Conclusions

In this study, we have proposed Deep4mcPred, a novel predictor for the prediction of DNA 4mC sites. Different from existing predictors using traditional machine learning algorithms (like SVM), Deep4mcPred is the first deep learning-based predictor, in which we integrate residual network and recurrent neural network–biLSTM to build a multi-layer deep learning predictive system. As compared to existing predictors, our proposed method has two advantages. First, our deep learning framework does not need to specify the features when training the predictive model. It can automatically learn the high-level features and capture the characteristic specificity of 4mC sites, benefiting to distinguish true 4mC sites from non-4mC sites. On the other hand, our deep learning method outperforms the traditional machine learning predictors in performance by benchmarking comparison. It demonstrates that the proposed Deep4mcPred is more effective in the DNA 4mC site prediction. Moreover, via experimental comparison, we found that attention mechanism introduced into the deep learning framework is useful to capture the critical features.

## Data Availability Statement

Publicly available datasets were analyzed in this study. This data can be found here: http://server.malab.cn/Deep4mcPred.

## Author Contributions

RZ wrote the manuscript, designed experiments, and did the results analysis. ML provided the idea.

## Conflict of Interest

The authors declare that the research was conducted in the absence of any commercial or financial relationships that could be construed as a potential conflict of interest.
